# Editorial: Non-Coding RNAs and Human Diseases

**DOI:** 10.3389/fgene.2020.00523

**Published:** 2020-05-25

**Authors:** Yujing Li, Ge Shan, Zhao-Qian Teng, Thomas S. Wingo

**Affiliations:** ^1^Department of Human Genetics, Emory University, Atlanta, GA, United States; ^2^CAS Key Laboratory of Innate Immunity and Chronic Disease, School of Life Sciences, University of Science and Technology of China, Hefei, China; ^3^State Key Laboratory of Stem Cell and Reproductive Biology, Institute of Zoology (CAS), Beijing, China; ^4^Department of Neurology, Emory University, Atlanta, GA, United States

**Keywords:** miRNA, ncRNAs, lncRNA, circRNA, piRNA, cancer biology, neurologcial disorders

Non-coding RNA (ncRNA) are functional RNA molecules that are not translatable into proteins (Djebali et al., 2012; Lonsdale et al., 2013; Forrest et al., 2014). Initially, ncRNAs referred to tRNAs and rRNAs (Brown et al., 1992; St Laurent et al., 2015). Recent technical advances have led to the discovery and characterization of many new classes of ncRNAs (Hüttenhofer and Vogel, 2006). These new ncRNAs species include snRNAs, snoRNAs, miRNAs, siRNAs, piRNAs, exRNAs, long non-coding RNA (lncRNAs), scaRNAs, and circRNAs (He and Hannon, 2004; Gu et al., 2007; Esteller, 2011; Redzic et al., 2014; Wu and Yang, 2015). While not all of their functions are known, many of the ncRNA species appear to play essential roles regulating transcription and translation of genes and transcription of ncRNAs themselves. Thus, there is little surprise that ncRNAs are identified as playing important roles in normal physiologic processes, complex human traits, and human diseases (Diederichs et al., 2016; Li et al., 2018; Fernandes et al., 2019; Vijayan and Reddy, 2020). This special issue focused on the ncRNA, particularly circRNAs, lncRNAs, miRNAs, and their role in human disease. The aim of this issue is to provide a broad overview of current research on the diverse work being done to elucidate the role of ncRNAs in disease. A major theme that emerged was the potential role of miRNAs as prognostic markers or biomarkers of disease.

## NcRNAs and Cancer

ncRNAs play vital roles in tumorigenesis and tumor progression that is incompletely understood. Several investigators addressed the role of circRNA-miRNA-mRNA networks in different cancers, including hepatocellular carcinoma (HCC) (Sheng et al.), gastrointestinal stromal tumors (Jia et al.), and cervical cancer (Liu C. et al.,). In a perspective, Molin et al. address the significance of circRNAs in MLL arranged acute leukemia (MLLre) recombinome. In lung adenocarcinoma, (Stewart et al.,) performed a large-scale analysis of lncRNAs and find evidence for deregulated pseudogene-derived lncRNAs associated with cancer survival. microRNAs are among the better known ncRNAs. Here, Pereira et al., identify miRNAs associated with the development of gastric cancer to novel targets and potential early-stage indicators. The role of miRNAs to identify HCC progression was also explored using fectal-derived miRNAs by Wang et al.. The role of miRNA and mRNA expression in endometrial cancer by Xu et al. using the The Cancer Genome Atlas (TCGA) identified mRNAs and miRNAs associated with patient survival suggesting a potential role for miRNAs in predicting clinical outcomes. The role of polymorphisms in miRNA and their contributions to cancer risk was explored by Choupani et al. by investigating the association of polymorphisms mir-196a-2 rs11614913 and mir-149 rs2292832 with multiple cancers (e.g., gynecological cancers, ovarian, breast, and HCC) in their updated meta-analysis. Finally, five review articles highlight the recent advances in the understanding of lncRNAs, circRNAs, and miRNAs in cancer initiation and progression, provide insight into their potential as biomarkers and therapeutic targets (Dong et al.; Plousiou and Vannini; Bandini and Fanini; Khan et al.).

To date, ncRNAs, particularly lncRNAs and miRNAs, are implicated in resistance or sensitivity to chemotherapy. Work by Xiang et al. analyzed the Cancer Cell Line Encyclopedia database and identified 44 of ncRNAs differentially expressed and significantly related to resistance or sensitivity of therapy for the advanced or metastatic breast cancer using Lapatinib, a small molecule inhibitor of HER1 and HER2 receptors. Shi et al. assayed miRNAs to identify the cisplatin-resistance in C13K human ovarian cancer cell and its cisplatin-sensitive OV2008 parental cells, and they found miR-205-5p led to PTEN downregulation and the subsequent enhancement of its downstream target p-AKT significantly contributes to cisplatin resistance in C13K Cells. Finally, Wan et al. addressed the cardiotoxicity from compound doxorubicin (DOX), a broad-spectrum anti-tumor drug. Their study found that the severe heart failure incurred by DOX based chemotherapy attributed to the enhanced expression of p21.

## NcRNAs and Liver and Cardiovascular Diseases

Non-alcoholic fatty liver disease (NAFLD) is a prevalent chronic liver condition that is associated with liver failure and HCC, Huang Z. et al. identified miRNAs that regulate the level of CYP3A4, an important drug-metabolizing enzyme associated with the progression of NAFLD, and they found that miR-200a-3p and miR-150-5p appear to directly regulate CYP3A4 and are involved in free fatty acid (FFA)-induced steatosis, implicating them in the NAFLD pathogenesis. The role of miRNAs was also examined in coronary artery disease (CAD), a prevalent human disease by Liu S. et al. who identified the miR-378a-5p-CDK1 axis as important in the proliferation and migration of vascular smooth muscle cells that can cause development of atherosclerosis and treatment failure for CAD.

## NcRNAs and Neurologic Disease

The role of lncRNAs to potentially address the wide-range of clinical severity and age-at-onset for spinocerebellar ataxia type 3, a rare neurodegenerative disease identified six lncRNAs that were initially identified in blood and then tested in cerebellum Li T. et al. In multiple sclerosis (MS), the most common chronic neurologic disease in young adults, identified circRNAs MS-associated genes and showed evidence that top MS-GWAS results are enriched for blocks with circRNAs thereby suggesting a potential novel role for circRNAs in MS pathogenesis (Paraboschi et al.). Review articles focused on recent progress of ncRNAs in many neurologic diseases including Alzheimer's Disease (Liu X. et al.), fragile X syndrome (Huang G. et al.; Zhou et al.), and neurodevelopmental disorders (Li L. et al.; Zhang et al.).

As the research and review articles of this issue highlight, ncRNAs are a diverse group of RNA species that are likely important contributors to human disease. They provide a unique window into disease pathogenesis and ability to target networks of genes and cellular processes. Future work to understand ncRNAs and their role in human illness will undoubtedly be aided by incorporating ncRNAs with transcriptomic and proteomic data. Such studies will be able to provide a more complete model of disease pathogenesis. We hope that future work will also examine the potential role of ncRNAs in prospectively collected samples from clinical trials or population-based samples to evaluate their suitability as prognostic indicators and biomarkers of disease.

## Author Contributions

YL, Z-QT, GS, and TW drafted the editorial. YL and TW revised the editorial with contributions from all authors. All authors approved the final version.

## Conflict of Interest

The authors declare that the research was conducted in the absence of any commercial or financial relationships that could be construed as a potential conflict of interest.
